# Impact of Theater Recovery Time on the Incidence of Postoperative Pneumonia in Patients Undergoing Major Lung Resection

**DOI:** 10.7759/cureus.85424

**Published:** 2025-06-05

**Authors:** Daniel Jones, Joshil Lodhia, Peter Tcherveniakov

**Affiliations:** 1 Trauma and Orthopaedics, Bradford Royal Infirmary, Bradford, GBR; 2 Thoracic Surgery, Leeds Teaching Hospitals NHS Trust, Leeds, GBR

**Keywords:** lung cancer, non-small cell lung cancer (nsclc), postoperative pneumonia, postoperative pulmonary complications, theatre recovery

## Abstract

Background and hypothesis

Postoperative pneumonia (POP) is a significant cause of morbidity and mortality following thoracic surgery and across various surgical specialties. We hypothesized that increased time spent in postoperative theater recovery may be associated with a higher incidence of POP and could serve as a potential marker for identifying vulnerable patients.

Method

Data on postoperative recovery time and subsequent POP diagnosis were obtained from an automated departmental database. A total of 577 patients who underwent lobectomy, bi-lobectomy, or pneumonectomy for non-small cell lung cancer between January 2019 and October 2021 were included in this study. A power calculation (beta = 0.2, power = 0.8) indicated a required sample size of at least 360 patients. Data analysis was performed using Microsoft Excel (Microsoft Corp., Redmond, WA, US) and IBM SPSS Statistics for Windows (IBM Corp., Armonk, NY, US).

Results

The data showed that patients diagnosed with POP spent a mean of 21 minutes 23 seconds longer in the theater recovery compared to those without POP; however, this difference did not reach statistical significance (p = 0.204). The distribution of recovery times was more skewed among POP patients (skewness = 3.68) compared to non-POP patients (skewness = 1.33). This prompted further analysis focused on patients who spent more than seven hours in postoperative recovery; again, no statistically significant difference was found between POP and non-POP patients (p = 0.224).

Conclusion

Although statistical significance was not demonstrated in this study, prolonged recovery time may still reflect an underlying vulnerability to POP, or it may simply represent inefficiencies in postoperative patient flow.

## Introduction

Postoperative pneumonia (POP) is a significant cause of morbidity and mortality in thoracic surgery and other surgical specialties [1-3]. It consistently ranks as the third most common postoperative infection after surgical site and urinary tract infections, with a reported mortality rate of 20%-50% [4-6]. Even among survivors, long-term outcomes are poor, with some studies reporting a 66% reduction in five-year survival following an episode of POP [7]. The incidence of POP following lung resection is not well known, with reported rates ranging from 2.1% to 29%. However, the most recent and robust study by Schussler et al. suggests an incidence of approximately 25% and an associated mortality rate of 19% [1,8,9]. In the context of thoracic surgery, diagnosing POP poses unique challenges. Postoperative fever, hypoxemia, and abnormal chest X-ray findings are common even in the absence of infection, complicating the identification of true cases of POP. Recognizing and modifying risk factors could play a key role in improving outcomes. Established risk factors for POP include advanced age, chronic obstructive pulmonary disease (COPD), current or former smoking, forced expiratory volume in 1 second (FEV1) < 70% of predicted, and male sex [10-12]. A comprehensive POP risk index has been developed using data from over 160,000 patients, yet it does not consider time spent in theater recovery as a potential risk factor, nor has it been explored in depth elsewhere in the literature [13]. Prolonged recovery time may reflect increased pain, higher oxygen requirements, or ineffective clearance of secretions (all risk factors for POP). We therefore aimed to investigate whether increased time in theater recovery is associated with an elevated incidence of POPP.

## Materials and methods

Study design

This was a retrospective cohort study. A power calculation (beta = 0.2, power = 0.8), based on data from Schussler et al. [1], indicated a required sample size of 360 patients. With 577 patients included in the analysis, the study was therefore overpowered.

Inclusion and exclusion criteria

Data from 588 patients who underwent lobectomy, bi-lobectomy, or pneumonectomy between January 2019 and October 2021 were analyzed. Eleven patients were excluded due to incomplete data, specifically missing time-in or time-out values from the recovery room. 

Data collection

Data collection was conducted via an automated system integrated with the electronic patient and theater records. The data were stored in an Excel database and managed under the supervision of the departmental data manager.

Data analysis

Statistical analyses were performed using Microsoft Excel (Microsoft Corp., Redmond, WA, US) and IBM SPSS Statistics for Windows (IBM Corp., Armonk, NY, US). Initial tests revealed that the data were not normally distributed and that variances were significantly unequal, as determined by Levene’s test. Consequently, the most appropriate method for assessing correlation was an independent samples t-test with bootstrapping.

## Results

**Figure 1:**
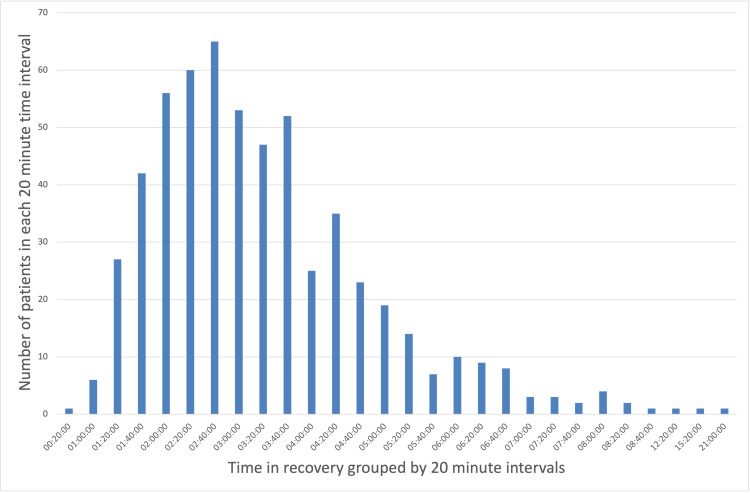
Distribution of time spent in recovery for all patients Bar chart illustrating the distribution of time spent in postoperative recovery by all patients who underwent lobectomy or pneumonectomy. Recovery times are grouped into 20-minute intervals. Here, the data are positively skewed (skewness = 3.07), with a long tail, and exhibit a leptokurtic distribution (kurtosis = 21.95).

**Figure 2:**
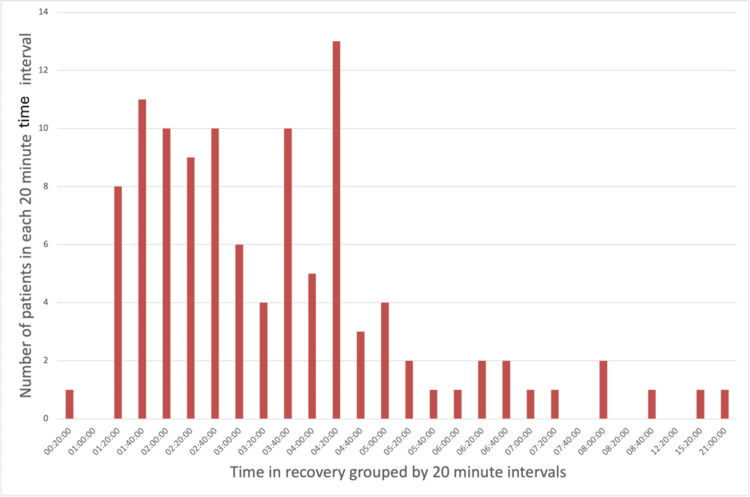
Distribution of time spent in recovery for patients who developed postoperative pneumonia (POP) Bar chart depicting the distribution of recovery times among patients who developed POP following lobectomy or pneumonectomy. Recovery times are grouped into 20-minute intervals. Here, the data are positively skewed (skewness = 3.68), with a long tail, and display a leptokurtic distribution (kurtosis = 20.10). Notably, the kurtosis is substantially higher than that observed in Figure 3, which represents patients who did not develop POP.

**Figure 3:**
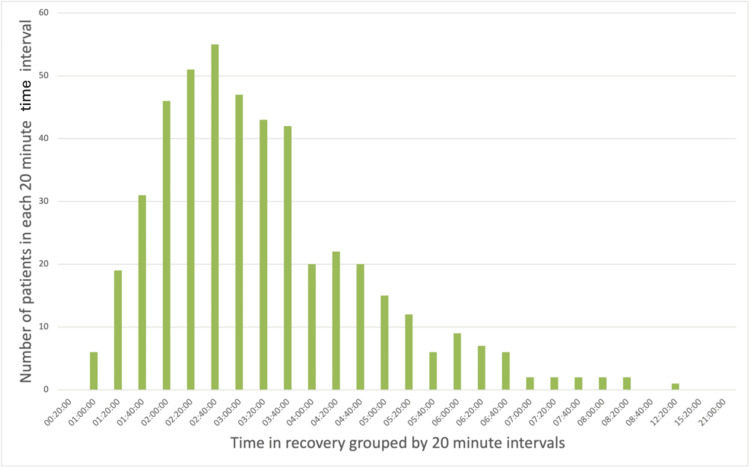
Distribution of time spent in recovery for patients who did not develop postoperative pneumonia (POP) Bar chart illustrating recovery time distribution among patients who did not develop POP following lobectomy or pneumonectomy. Recovery times are grouped into 20-minute intervals. Here, the data are positively skewed (skewness = 1.33), with a long tail, and exhibit a slightly leptokurtic, or approximately mesokurtic, distribution (kurtosis = 3.25). Notably, the kurtosis is substantially lower than that observed in patients who developed POP, as shown in Figure 2.

Data on the incidence of POP among the included patients are summarized in Table 1. Of the 577 patients analyzed, 109 (18.9%) developed POP. These patients spent an average of 21 minutes and 23 seconds longer in recovery compared to those who did not develop POP. To visually illustrate the distribution of recovery times, the data were grouped into 20-minute intervals and are presented in Figures 1-3. An independent samples t-test with bootstrapping was used to assess the difference in recovery times. Although patients who developed POP spent more time in recovery on average, this difference was not statistically significant (p = 0.204). 

**Table 1:** Incidence and recovery time comparison for patients with and without postoperative pneumonia (POP)

Total number of patients	577
Total cases of POP	109
Percentage of patients with POP	18.9%
Average time (hours:minutes:seconds) in recovery for patients with a POP diagnosis	03:49:51
Average time (hours:minutes:seconds) in recovery for patients without a POP diagnosis	03:28:28
Difference in recovery time between patients with and without a POP diagnosis	00:21:23
Skew for all patients	3.07
Kurtosis for all patients	21.95
Skew for patients with a POP diagnosis	3.68
Kurtosis for patients with a POP diagnosis	20.10
Skew for patients without a POP diagnosis	1.33
Kurtosis for patients without a POP diagnosis	3.25
Independent samples t-test with bootstrapping p-value	0.204 (sig = 0.05)

Data presented in Table 2 comprises a secondary analysis of patients who spent more than seven hours in postoperative recovery. This subgroup analysis was prompted by the observation of markedly greater skewness in the recovery time data for patients who developed POP. As with the primary analysis, the data were non-normally distributed and demonstrated significantly unequal variance. Consequently, an independent samples t-test with bootstrapping was used. The results again did not demonstrate statistical significance (p = 0.224).

**Table 2:** Postoperative pneumonia (POP) incidence among patients spending more than seven hours in theater recovery

Total patients (with ≥ 7 hours in postop recovery)	45
Total cases of POP (with ≥ 7 hours in postop recovery)	12
Percentage of patients with POP (and ≥ 7 hours in postop recovery)	26.67%
Average time (hours:minutes:seconds) in recovery for patients with a POP diagnosis (and ≥ 7 hours in postop recovery)	09:05:13
Average time (hours:minutes:seconds) in recovery for patients without a POP diagnosis (and ≥ 7 hours in postop recovery)	07:02:02
Skew for all patients (and ≥ 7 hours in postop recovery)	3.87
Kurtosis for all patients (and ≥ 7 hours in postop recovery)	16.71
Skewness for patients with a POP diagnosis (and ≥ 7 hours in postop recovery)	2.21
Kurtosis for patients with a POP diagnosis (and ≥ 7 hours in postop recovery)	4.42
Skewness for patients without a POP diagnosis (and ≥ 7 hours in postop recovery)	3.03
Kurtosis for patients without a POP diagnosis (and ≥ 7 hours in postop recovery)	12.26
Independent samples t-test with bootstrapping p-value (and ≥ 7 hours in postop recovery)	0.224 (sig = 0.05)

## Discussion

These data indicate a positive correlation: patients who developed POP spent a mean of 21 minutes and 23 seconds longer in recovery. However, this difference did not reach statistical significance (p = 0.204). A comparison of kurtosis values across groups, POP (20.10), non-POP (3.25), and the overall dataset (21.95), suggests that a disproportionate number of outliers are contributed by patients who developed POP. While this is a notable finding, further analysis of the subgroup of patients who spent ≥ 7 hours in postoperative recovery also failed to demonstrate statistical significance (p = 0.224). It is important to note that this subgroup represented a relatively small sample size, and a larger study would be necessary to determine whether a true association exists.

A previous retrospective study involving 466 patients reported a 10-fold increase in the risk of POP when comparing lobectomy with bi-lobectomy [12]. Further stratified analysis of our dataset could explore recovery room durations across different surgical procedures (pneumonectomy, bi-lobectomy, and lobectomy), to assess whether procedure type influences time in recovery and associated POP risk. Additionally, surgical approach may play a role; thoracotomy, in contrast to video-assisted thoracic surgery (VATS), has been associated with greater postoperative immunosuppression, increased pain, and a higher risk of POP [14-16]. The choice of analgesia could also be accounted for. Thoracic epidural block (TEB) and paravertebral block (PVB) are both widely used in thoracic surgery. At our center, PVB is preferred over TEB due to its reduced risk of minor complications such as hypotension, pruritus, and retention [17]. Notably, the timing of PVB, whether applied early at the start of surgery or later before closure, may influence outcomes. Some evidence suggests early application reduces postoperative opioid requirements, and higher systemic opioid use has been linked to increased POP incidence [18,19]. Ultimately, a more comprehensive analysis using propensity score matching in a larger population could better account for these variables, including patient age, comorbidities, and smoking status.

Air quality in postoperative recovery rooms may also represent a valuable avenue for future research into POP. A bacterial air concentration analysis conducted in a Taiwanese hospital found that the postoperative recovery room had the highest bacterial concentration compared to operating theaters, supply rooms, and even hospital restaurants [20]. Similarly, a Thai study reported airborne bacterial concentrations in postoperative recovery rooms exceeding recommended UK guidelines [21], though, notably, neither study examined bacterial air concentrations on wards. However, a German investigation further highlighted that airborne contamination in hospital wards and postoperative recovery rooms was greater than in homes within the same community [22]. The applicability of these findings to UK settings may be limited due to geographical and infrastructural differences. Therefore, any future UK-based study could benefit from including air quality assessments specific to the setting. The clinical relevance of air quality could be investigated by analyzing POP incidence among patients who bypass the postoperative recovery room. Bypassing the postoperative recovery room may occur due to logistical reasons, such as bed unavailability, insufficient staffing, or closure of recovery rooms during late cases, or due to infection control protocols, such as for patients colonized with methicillin-resistant *Staphylococcus aureus* (MRSA) or carbapenemase-producing Enterobacteriaceae (CPE), who may recover in theater before direct transfer to isolated ward rooms. These variables are not currently captured in the departmental database, but could be included in a prospective study. Another potential factor worth exploring is the "traffic flow" within postoperative recovery rooms. Increased door openings and staff movement have been shown to increase airborne bacterial loads in operating theaters and likely have a similar impact on postoperative recovery [23].

A limitation of this study is the potential for misdiagnosis of POP. Identifying consolidation amid postoperative chest X-ray changes can be challenging, many patients do not return positive sputum cultures, and clinical features of pneumonia can overlap with postoperative inflammation. Consequently, mild cases may go undiagnosed in hospitals and be managed later in the community post-discharge by general practitioners. Conversely, patients perceived to be at higher risk may receive treatment for presumed POP despite limited radiological or clinical evidence, potentially leading to an overestimation of the incidence of POP. A future prospective study with standardized diagnostic criteria for POP would help mitigate this bias and provide more accurate incidence data.

## Conclusions

Extended time in postoperative recovery is unlikely to be a direct cause of POP; rather, it may serve as a marker to identify patients vulnerable to clinical deterioration. As described earlier, in some patients, prolonged recovery time may reflect underlying physiological instability, manifested as reduced Glasgow Coma Scale (GCS) scores or prolonged periods of assisted ventilation, which increases the risk of POP through poorer control of swallowing and secretions. In other cases, however, increased recovery time may result from non-clinical factors such as delayed review of postoperative imaging, bed unavailability, or other inefficiencies in patient flow. Nevertheless, though recovery time is perhaps not a decisive marker for patients vulnerable to POP, it is still pertinent to take additional note of patients who have an extended recovery stay and to interpret it in the context of their overall clinical picture.
